# Quantitative detection of pseudouridine in RNA by mass spectrometry

**DOI:** 10.1038/s41598-024-78734-3

**Published:** 2024-11-11

**Authors:** Shanice Jessica Hermon, Anastasia Sennikova, Sidney Becker

**Affiliations:** 1 Max-Planck Institute of Molecular Physiology, 44227 Dortmund, Germany; 2 Department of Chemistry and Chemical Biology, Technical University Dortmund, 44227 Dortmund, Germany

**Keywords:** RNA modifications, Mass spectrometry, Pseudouridine, tRNA, Bioanalytical chemistry, Nucleic acids, RNA, Mass spectrometry

## Abstract

Pseudouridine (Ψ) is one of the most prevalent and dynamic modification in RNA, and was shown to evade the host immune response in mRNA vaccines. Despite its significance, the biological role of Ψ remains poorly understood as certain key limitations and challenges in the detection of Ψ are yet to be overcome. In account of this, we report the usage of a chemical labelling strategy for the first quantitative detection of Ψ by mass spectrometry. We demonstrate a labelling efficiency exceeding 99% in isolated yeast tRNAs hosting multiple Ψs. LC-MS/MS analysis enables precise mapping of Ψ at single-base resolution, while simultaneously capturing a wide array of additional post-transcriptional modifications, which is not achieved with current sequencing technologies. This advancement may help unravel the dynamics and biological implications of Ψ, shedding light on its interplay with other modifications and deepening our understanding of its functional role.

**Supplementary Information:**

The online version contains supplementary material available at 10.1038/s41598-024-78734-3.

## Introduction

Post-transcriptional modifications are involved in fundamental cellular processes. They modulate gene expression by fine-tuning transcription, RNA processing and protein synthesis, thereby establishing multiple regulatory layers. Therefore, their spatiotemporal deposition is vital for normal human development and dysregulation is connected to pathology. So far, however, the functional implications of most RNA modifications are largely elusive. More than 150 different modifications have been discovered in both coding and noncoding RNAs^1^. They alter the physicochemical properties of nucleic acids to influence RNA structure, function and stability^1,2^.

Pseudouridine (Ψ) is one of the most prevalent and dynamic modification, found in many functional RNA species including tRNAs, rRNAs and snRNAs^3–5^. Ψ is installed by the pseudouridine synthases (PUS), from isomerization of uridine, which creates an additional hydrogen bond donor at the N1-position^3^. These structural changes improve base stacking and stabilize the folded RNA structure^6^. Ψ is believed to be involved in regulating translation^5,7^, splicing^5,8^, RNA–protein interactions^5^ and in modulating environmental stress response^3,9^. Investigating Ψs distribution, dynamics and interdependency with other epitranscriptional modifications will help to provide a better mechanistic understanding to uncover its exact biological role. This, however, requires reliable detection methods at single base resolution.

Current approaches to map Ψ are mainly based on mass spectrometry (LC–MS) or next generation sequencing (NGS) technologies, both of which are unable to directly detect Ψ^10^. Consequently, NGS methods mostly utilize chemical labelling of Ψ followed by its indirect detection from deletion signatures from reverse transcription. Ψ detection, however, varies significantly between the different NGS methods and shows strong sequence context dependency, causing limited overlap of the identified Ψ-sites^11^. Moreover, as NGS methods follow an indirect mode of detection, the extend of false positives/negatives are high, especially if Ψ-sites are near uridines^12–14^. These methods cannot provide quantitative estimates of Ψ at a given site. Additionally, as NGS methods can only analyze a limited number of modifications in parallel, the effect of these methods on other RNA modifications is therefore not well established. These concerns have led to the recent developments of nanopore sequencing which identifies Ψ sites as high confidence U to C base calling errors^15–19^. Many of these methods, however, cannot truly discriminate Ψ from other U-derived modifications such as Dihydrouridine (D) and 2’-O-methyluridine (Um)^15^. To overcome these limitations, subsequent approaches utilize chemically assisted nanopore sequencing for the detection of Ψ as in-del signatures^20^. Nevertheless, the sequence context dependency of Ψ in base calling and the occurrence of false positives inevitably lead to high error rates in nanopore sequencing methods^16–20^.

As an orthogonal strategy, mass spectrometry-based analysis is a powerful tool that allows for the direct, sequence independent detection of multiple modifications in parallel, thereby significantly reducing potential biases. As Ψ exhibit the same mass as uridine, most mass spectrometry-based methods rely on chemical derivatization strategies to generate a mass tag on Ψ for its detection. A widely used method is the chemical derivatization with CMC (N-cyclohexyl-N′-β-(4-methylmorpholinium) ethylcarbodiimide), which generates a mass tag of 252 Da^21^, while acrylonitrile provides a mass difference of 53 Da^22–24^. However, the labelling efficiency of these reported methods is significantly low, leading to the loss of valuable quantitative information. In addition, false positives are observed due to uridine labelling. CMC is also reported to label 2-methylthio-N6-isopentenyladenosine^25^, complicating analysis. Alternatively, stable isotope labelling for Ψ quantification^26^ and characteristic Ψ-specific collision induced dissociation (CID) pattern for Ψ-detection are also reported^27,28^. These methods, however, can reliably detect Ψ only in short RNA sequences in the absence of neighboring uridines.

**Fig. 1:**
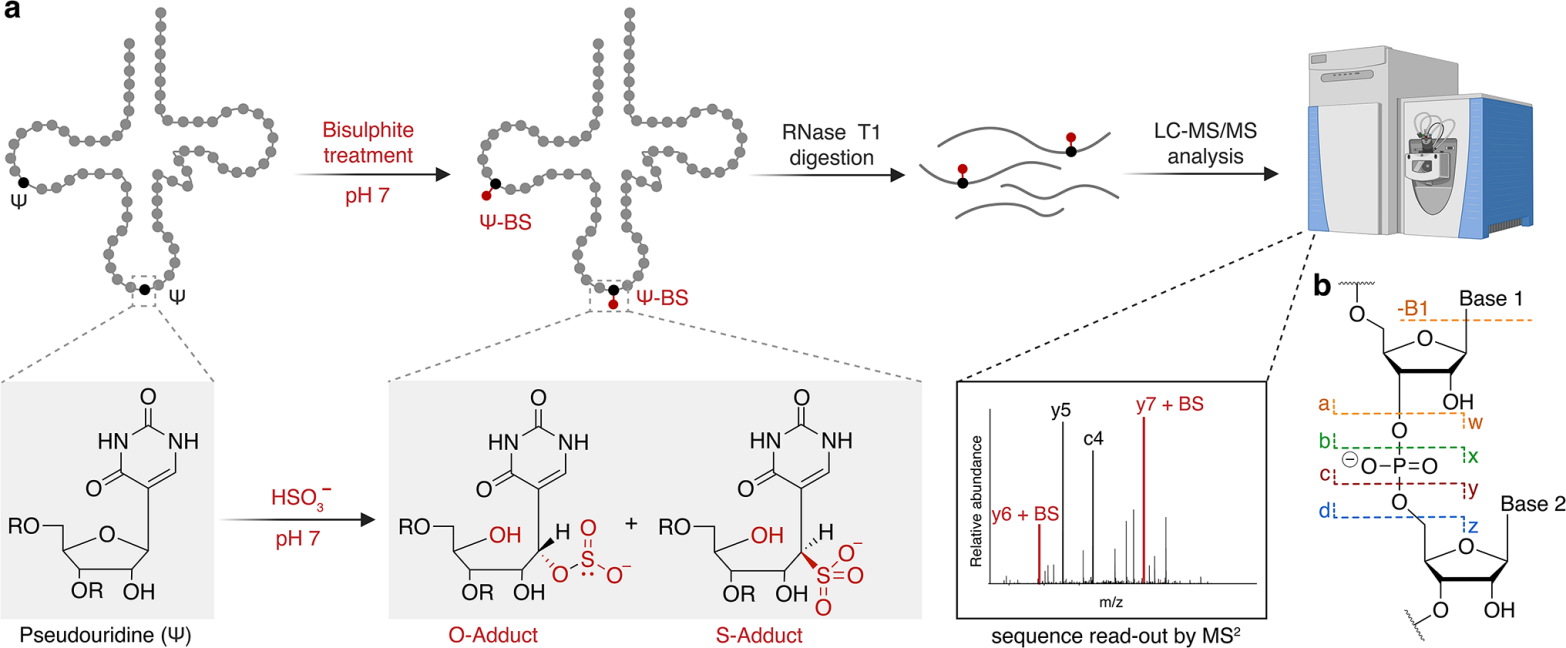
Overview of the bisulphite labelling strategy used for the LC–MS/MS detection of pseudouridine (Ψ) in tRNA. (**a**) Bisulphite labelling of Ψ in tRNA generates either the S adduct or the O adduct, both of which are indistinguishable in LC–MS/MS analysis. Following labelling, the tRNAs are treated with RNase T1 to generate smaller fragments for analysis. In the MS/MS analysis, all CID fragments which hosts the Ψ-BS adducts show a mass shift of 82 Da, enabling the identification of Ψ in the sequence. (**b**) CID fragmentation pattern of RNA producing 9 major fragment ion series (*a, a-B, b, c, d, w, x, y, and z*). Part of the figure is created with BioRender.com.

The key limitations and challenges that are yet to be overcome calls for the development of efficient methods for both qualitative and quantitative Ψ-detection. The parallel read-out of various modifications concurrently can shed light on Ψs interplay with other post-transcriptional RNA modifications. Therefore, we considered potential Ψ labelling reagents that could provide quantitative sequence read-out by mass spectrometry. A promising candidate is bisulphite (BS), which is known to convert cytosine to uridine (C-to-U) at acidic pH. Bisulphite reacts covalently with Ψ as well, attaching to the C1’ of the ring opened ribose via the sulphur (S adduct) or oxygen (O adduct) atom of the bisulphite nucleophile (Fig. 1a). This reaction has been applied in recent NGS methods for the indirect detection of Ψ^29–32^. If the reaction is performed at neutral pH, labelling of Ψ occurs without C-to-U conversion. Slightly basic pH treatment reverses any unspecific bisulphite label on uridine or cytosine nucleosides^30,33^.

Thus, we explored the specificity and efficiency of bisulphite labelling on Ψ-containing RNA oligonucleotides. We further evaluated if the labelling could be exploited for the efficient and quantitative mass spectrometry-based detection of Ψ. For this, we first optimized labelling and sample preparation procedures as well as LC–MS/MS methodologies on RNA oligonucleotides. Upon comparison with existing LC–MS based Ψ-labelling methods, we found that bisulphite labelling shows superior selectivity and efficiency, which allows for the first quantitative detection of Ψ at single base resolution. We further show the compatibility of bisulphite labelling within biological tRNA samples. Isolating tRNA^His^, tRNA^Leu^ and tRNA^Arg^ from yeast tRNA extract allowed for LC–MS analysis of the intact tRNAs. RNase T1 digestion and subsequent LC–MS/MS analysis of tRNA^His^ and tRNA^Leu^, enabled the identification of the exact sequence position of bisulphite labelled Ψ in parallel with all other RNA modifications that are present in the mentioned tRNAs.

## Results

### Detection and quantification of Ψ in synthetic RNA oligonucleotides

For initial trials, we tested the efficiency of bisulphite labelling (pH 7) on synthetic 30-mer RNA oligonucleotides with one or more Ψs in various sequence contexts. As negative control, we performed labelling on the exact same RNA oligonucleotide sequence with uridine (U) instead of Ψ. Following labelling, the samples were subjected to slightly basic pH conditions (pH 9) for desulphonation to remove any non-specific label. Omitting this step results in in the detection of false positives (Supplementary Fig. S1). We further confirmed that no degradation of the RNA oligonucleotides is observed after bisulphite treatment (Supplementary Fig. S2).

**Fig. 2:**
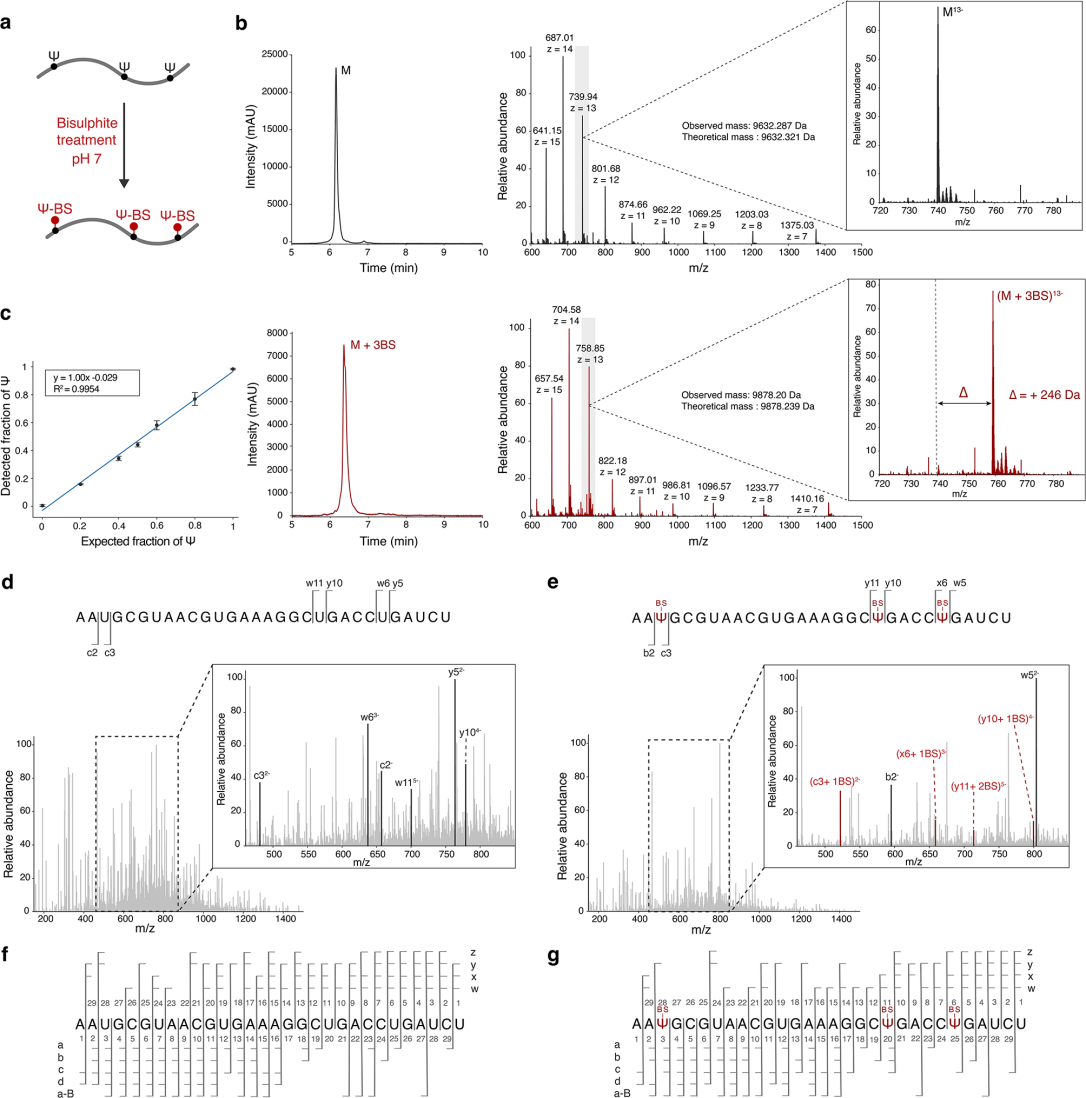
LC–MS/MS analysis of bisulphite treated synthetic RNA oligonucleotides. (**a**) Schematic representation of the bisulphite treatment of synthetic RNA oligonucleotides RNA1U and RNA1P. (**b**) top: HPLC profile (left) and MS1 spectra (right) of the control RNA oligonucleotide (RNA1U) devoid of Ψ, following treatment with bisulphite. The multiple charged negative ion series of the intact RNA1U is shown. The mass peak of 739.94, with a charge state of -13 (M^13-^) shown in the inset. bottom: HPLC profile (left) and MS1 spectra (right) of the RNA oligonucleotide hosting three Ψs in parallel (RNA1P), following treatment with bisulphite. The multiple charged negative ion series of the bisulphite labelled intact RNA1P is shown. The mass peak of 758.85, with a charge state of -13 ((M + 3BS)^13-^) is shown in the inset. The mass peak of the labelled RNA1P shifts from the control RNA1U (as indicated by the dotted line) by 246 Da. (**c**) Quantification of Ψ from sample mixtures of control RNA2U and RNA oligonucleotide with a single Ψ (RNA2P) in variable ratios. The sequences of the control RNA-RNA1U and the RNA with Ψ (RNA2P) is GUUCCCAUCCGUAGCUA**U**GAAGACGUGCGU and GUUCCCAUCCGUAGCUA**Ψ**GAAGACGUGCGU respectively. A fitting curve is plotted based on the observed Ψ fraction and the expected Ψ fraction. (**d**, **e**)**,** CID spectra of the control (RNA1U) and the RNA with Ψ (RNA1P) treated with bisulphite (sequences mentioned at the top). The precursor ions for fragmentation are M^13-^ and (M + 2BS)^13-^ respectively. Assignment of the CID fragment ions reveal the positions of the Ψ at 3, 20 and 25 as expected. (**f, g**) Representation of all fragment ions identified in both RNA1U and RNA1P following BS treatment.

In all tested Ψ-containing RNAs, we observed quantitative labelling regardless of the sequence context or number of Ψs (Fig. 2, Supplementary Figs. S3–S5). The bisulphite label introduces a mass tag of 82 Da onto Ψ, which is stable under ESI–MS conditions and can be detected by intact mass analysis. The use of an RNA oligonucleotide containing three Ψs shows complete labelling with a mass shift of 246 Da (3 × 82 Da), confirming the presence of the expected number of Ψs. In contrast, the mass of the control oligonucleotide (where Ψ is replaced by U) remained unchanged following bisulphite treatment (Fig. 2a,b). This confirmed the high specificity of the label. We further tested for C-BS and U-BS adduct formation in the currently optimized conditions. For this, the bisulphite labelling procedure was tested on multiple RNA oligonucleotides with varying sequence contexts of U and C. Nonspecific labelling was not observed (Supplementary Fig. S6). In addition, no cytosine deamination was detected under neutral bisulphite treatment (pH 7). It is known, however, that C-to-U conversion takes place at lower pH^34^. Therefore, as a positive control we confirmed cytosine deamination with a commercially available RNA bisulphite kit that uses acidic pH conditions (Supplementary Fig. S6).

To verify if the bisulphite labelling is indeed quantitative, we tested the labelling efficiency in mixed oligonucleotide samples containing different ratios of the Ψ-containing RNA vs. control sequence. The samples were treated with bisulphite and the intact mass of the labelled and unlabeled sequences were quantified by signal integration. The expected fraction was plotted against the measured fraction (Supplementary Table S1). Fitting a linear regression provided a slope of 1.00, confirming highly specific and quantitative Ψ labelling. Indeed, the experimental values showed a standard deviation < 6% (from three replicates) for all tested fractions (Fig. 2c). This confirmed that bisulphite labelling at neutral pH enables robust and quantitative detection of Ψ without effecting other canonical bases in the RNA.

Next, we confirmed the exact sequence position of Ψ. For this, we first optimized MS parameters for collision induced dissociation (CID) of RNA oligonucleotides, to obtain full sequence coverage (Supplementary Fig. S7). CID generates *a/a-B, b, c, d, w, x, y and z* type ions in a ladder-like fashion, with the *c* and *y* ions most common for RNA^35,36^ (Fig. 1b). As Ψ and U containing RNA oligonucleotides cannot be discriminated by intact mass, the fragment ions generated upon CID are also identical. Ψ-labelling with bisulphite will not only change the intact mass but also create altered fragment ions upon CID fragmentation. After optimizing MS-parameters, we confirmed that the bisulphite label is stable under these MS/MS conditions to identify its exact sequence position (Fig. 2d). The control oligo (without Ψ) does not have an identifiable label in any of the identified fragments (Fig. 2e). In the labeled oligo (with Ψ), the fragments *c3, y11* and *x6* differs from *b2, y10* and *w5*, by an additional mass of the Ψ-BS adduct (+ 82 Da), perfectly demarcating the position of Ψ in the sequence (Fig. 2d,e). Overall, all 30-mer RNA oligonucleotides showed 100% sequence coverage and stable bisulphite labelling upon optimized CID fragmentation, allowing to obtain the exact Ψ position within the sequence (Fig. 2f,g, Supplementary Figs. S3–S5).

### Comparison with reported labelling strategies

**Fig. 3:**
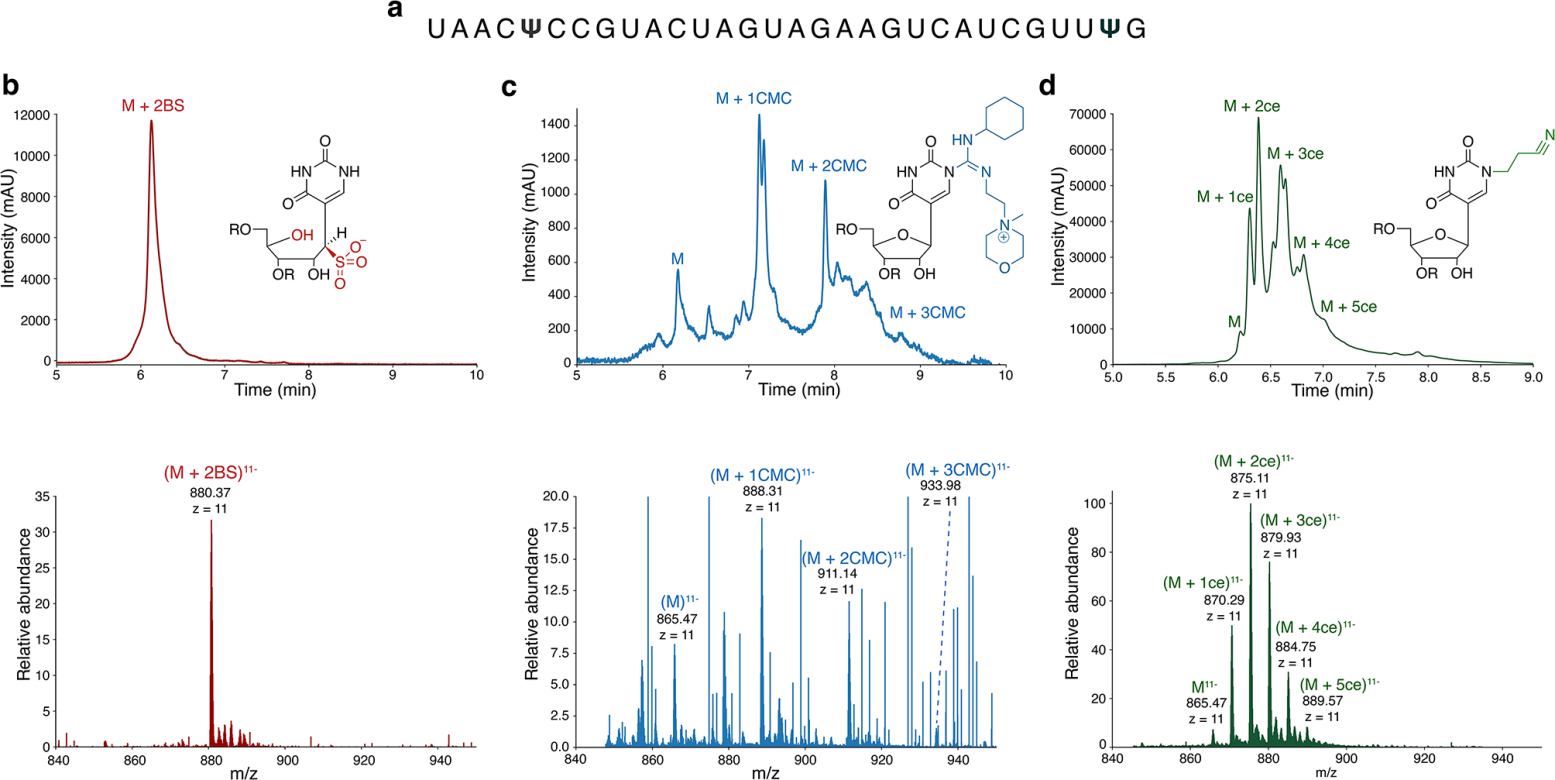
Comparison of the specificity and efficiency of labelling Ψ by CMC, acrylonitrile and bisulphite. (**a**) RNA sequence with two Ψs in parallel (RNA3P) used for all three labelling studies. (**b**) HPLC profile of the bisulphite labelled RNA3P showing a single clean peak and the chemical structure of Ψ-BS adduct (top). MS1 spectra of the BS labelled RNA3P showing a single clean peak with a mass of 880.37, with a charge state of -11(bottom). (**c**) HPLC profile of the CMC labelled RNA3P, and the chemical structure of the CMC labelled Ψ (top). MS1 spectra of the CMC labelled RNA3P, the mass peak of the intact mass of the RNA3P is labelled as M^11-^, CMC labelled peaks are represented as (M + n CMC)^11-^, where n = number of CMC labels on the RNA. **d,** HPLC profile of the cyanoethylated (ce) RNA3P, and the chemical structure of the acrylonitrile labelled Ψ. (top). MS1 spectra of the acrylonitrile labelled RNA3P, the mass peak of the intact mass of the RNA is labelled as M^11-^, cyanoethylated peaks are represented as (M + n ce)^11-^, where n = number of acrylonitrile labels on the RNA.

Next, we tested how the bisulphite labelling compares to previously reported Ψ-labelling methods. Of these, the CMC labelling strategy and cyanoethylation are the most commonly applied methods^21,24,25^. Both methods have contributed significantly in the detection of novel Ψ sites^37^. In account of this, we compared the efficiency and specificity of bisulphite, CMC and acrylonitrile derivatization for LC–MS detection of Ψ (Fig. 3).

We first treated an oligonucleotide carrying two Ψs with bisulphite and observed a single mass peak. The peak corresponds to a mass shift of 164 Da from the unlabeled intact mass, confirming a complete conversion of Ψ to Ψ-BS adducts. No false positives or partially labelled species were observed upon bisulphite treatment (Fig. 3b).

Next, we performed CMC labelling on the same oligonucleotide (containing two Ψs), using reported experimental conditions^37–39^. Derivatization with CMC modifies the N1 or N3 of Ψ, N1 of guanine and N3 of uridine. Further treatment at a pH of 10.4 for 4 h removes the CMC from guanine and uridine, maintaining the specificity to Ψ^25^. This generates a Ψ-mass tag of 252 Da, enabling its discrimination from uridine. Following CMC treatment, we observe the labelling to be incomplete as multiple LC–MS peaks are identified corresponding to unlabeled, single labelled and double labelled oligonucleotide. A triple labelled specie suggests formation of false positives (Fig. 3c). Even though CMC is widely used, this method suffers from several limitations including potential false negatives/positives due to low labelling efficiency and specificity^25^. The high pH conditions for reversing unspecific CMC labelling create multiple degradation products, thus reducing overall data quality (Fig. 3c).

Lastly, we tested acrylonitrile labelling of Ψ, which generates 1-cyanoethyl-Ψ with a mass shift of 53 Da from unlabeled Ψ^24^. Upon treatment with acrylonitrile following a reported procedure^24^, multiple species with two, three, four and five labels, differing by a mass of 53 Da were observed (Fig. 3d). This suggests that the method generates significant false positives. A reason could be the nonselective derivatization of uridine^23^. In addition, non-labelled as well as single labelled oligonucleotides are observed, which demonstrates low labelling efficiency.

We repeated the three labelling reactions (bisulphite, CMC and acrylonitrile) on another oligonucleotide to confirm that the results are reproducible (Supplementary Fig. S8). Overall, our experiments suggest that bisulphite labelling surpasses existing mass spectrometry based pseudouridine detection strategies in terms of efficiency and specificity. This allows for the first direct and quantitative read-out of Ψ in RNA.

### Detection of Ψ in isolated yeast tRNAs

**Fig. 4:**
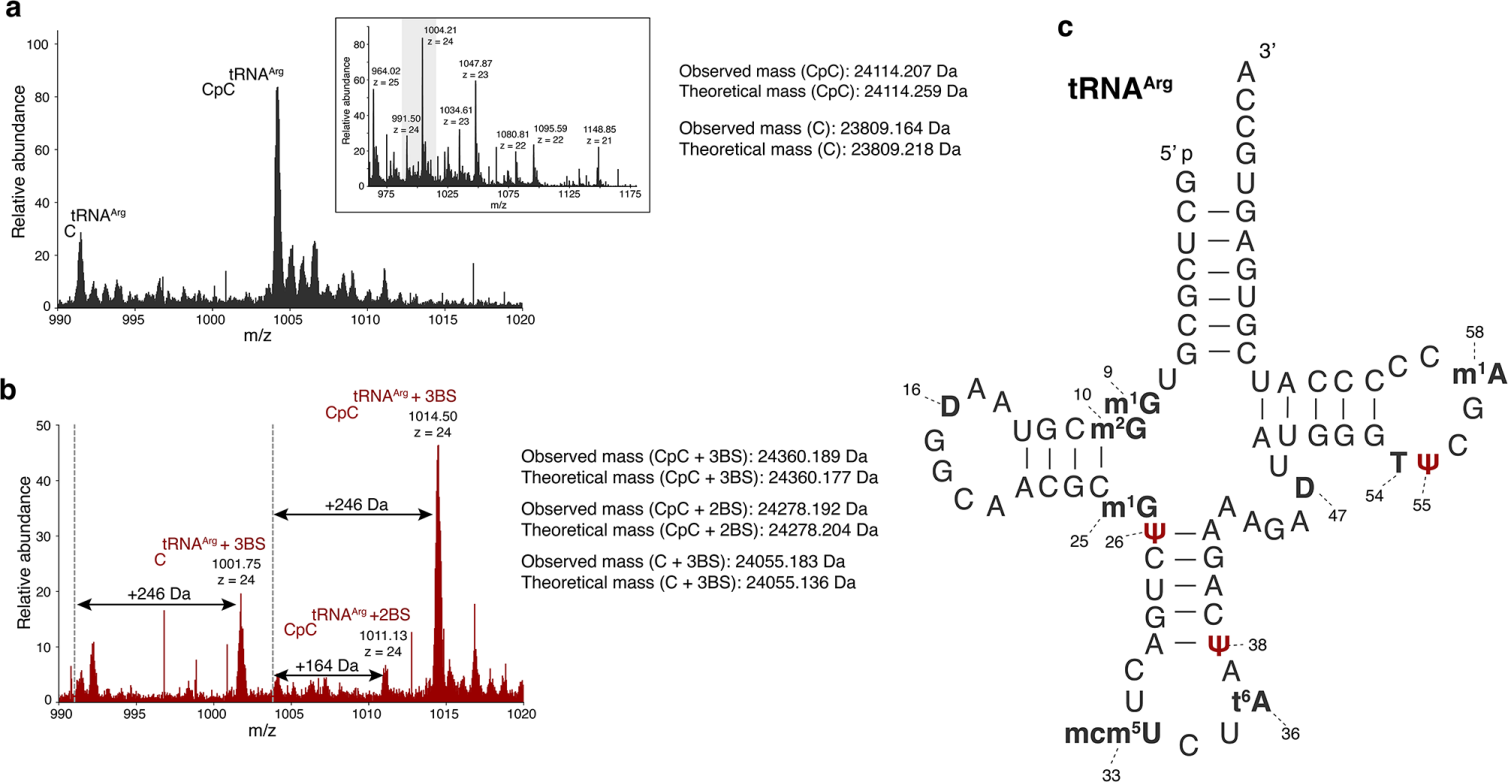
LC–MS analysis of bisulphite labelled tRNA^Arg^ (**a**) LC–MS analysis of the intact tRNA^Arg^. The mass peaks of 1004.21, and 991.50 with charge states of -24, of the unlabeled intact tRNA is shown. The series of multiply charged negative ions of the intact tRNA^Arg^ is shown in the inset. (**b**) LC–MS analysis of the bisulphite labelled intact tRNA^Arg^, the labelled mass peaks, with charge states of -24 is shown. The mass peak of the labelled tRNA^Arg^ shifts from the unlabeled one (as indicated by the dotted line) by 246 Da or 164 Da. (**c**) Secondary structure of yeast tRNA^Arg^ (database ID: tdbR00000370)^40,41^. The positions of all modified residues are marked. The symbols for the modified nucleosides are as follows: m^1^G, 1-methylguanosine; m^2^G, 2-methylguanosine; Ψ, Pseudouridine; mcm^5^U, 5-methoxycarbonylmethyluridine; t^6^A, N6-threonylcarbamoyladenosine; D, Dihydrouridine; T, Thymidine; m^1^A, 1-methyladenosine.

Finally, we performed labelling on biologically relevant tRNA samples, which are heavily modified. Therefore, the effect of bisulphite labelling on other RNA modifications can be assessed. In addition, it allows for the base resolution read-out of Ψ in parallel with all other post-transcriptional tRNA modifications. We used yeast tRNA extract and analyzed its composition by LC–MS to identify the most abundant tRNA species for isolation. Using biotin labelled DNA oligonucleotide probes targeting the respective tRNAs, we performed streptavidin-based pull down to isolate tRNA^His^, tRNA^Leu^ and tRNA^Arg^ from a yeast tRNA mixture. The identity of the isolated tRNAs were confirmed by LC–MS analysis by comparing the respective masses of the tRNA variants (3’-end: CCA, CC or C) to the masses of the sequences reported in the database^40,41^. All tRNA variants were identified with an error of less than 3 ppm (Fig. 4, Supplementary Figs. S9,S10).

Most tRNA modifications are methylations such as m^5^C, m^1^G, m^2^G, m^22^G, Am, which are not expected to be altered by bisulphite (Fig. 4, Supplementary Figs. S9, S10). The three isolated tRNAs (tRNA^His^, tRNA^Leu^ and tRNA^Arg^) also host dihydrouridines (D) at multiple sequence positions and thymine in the T loop (Fig. 4, Supplementary Figs. S9, S10). In addition, tRNA^Arg^ contains mcm5U (5-methoxycarbonylmethyluridine) and t6A (N6-threonylcarbamoyl-adenosine) (Fig. 4)^40,41^ . Upon bisulphite labelling, the intact mass analysis of all three tRNAs suggests the presence of three Ψs in each of the sequences, as reported in the tRNA database (Fig. 4, Supplementary Figs. S9,S10). For tRNA^His^, we observed a complete conversion from the unlabeled to the triple labelled specie (Supplementary Fig. S9). In contrast, tRNA^Arg^ provides two differently labelled species, hosting either two Ψs (7.82%) or three Ψs (92.18%). In tRNA^Leu^, we assumed the loss of an acetyl group (from ac^4^C) since the mass difference was only 204 Da (3BS – ac) instead of the expected 246 Da (Supplementary Fig. S10). However, all other modifications present in tRNA^His^, tRNA^Leu^ and tRNA^Arg^ were not affected by the labelling.

**Fig. 5:**
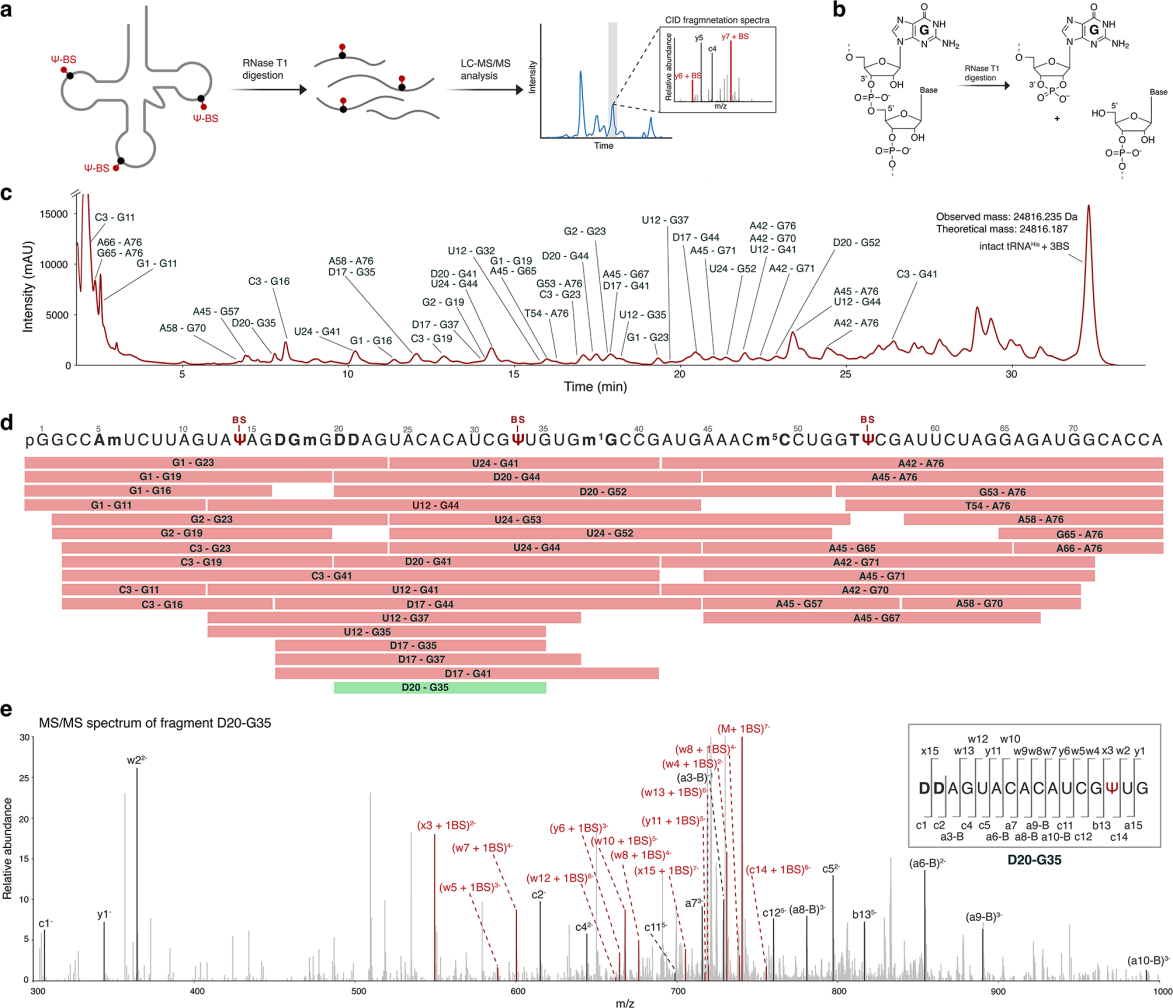
LC–MS/MS analysis of bisulphite labelled tRNA^His^ following digestion with RNase T1. (**a**, **b**) Schematic representation of RNase T1 digestion of tRNA and LC–MS/MS analysis. RNase T1 cleaves after guanosine, generating 5’-OH at the adjacent nucleoside residue and 2′3’-cyclic phosphate termini at the guanosine^45^. (**c**) HPLC profile of the RNase T1 digested fragments of the bisulphite labelled tRNA^His^, all identified fragments are labelled. (**d**) Sequence map showing all MS/MS identified RNase T1 digestion fragments from the bisulphite labelled tRNA^His^. Only fragments with a 2′3’-cyclic phosphate, full structural resolution and error < 5 ppm are considered for sequence mapping. The sequence map covers 100% of the tRNA^His^ sequence identifying all expected modifications including labelled Ψ. (**e**) CID spectra of the fragment D20 – G35 (green fragment in **d**). The precursor ion for fragmentation is (M + 1BS)^7-^.

We finally verified that our bisulphite label is found in the expected Ψ-positions and further profiled all other post-transcriptional modifications within tRNA^His^ and tRNA^Leu^. The LC–MS/MS analysis of full length tRNA is currently problematic due to technical limitations such as sensitivity. Therefore, we performed a partial digestion by RNase T1 to obtain shorter RNA sequences. Chromatographic separation and CID-fragmentation allowed us to accurately read the full tRNA sequence including its modifications (Fig. 5a,b). For analysis, we only considered the MS/MS data of RNase digested fragments with an error of less than 10 ppm. For unlabeled and labelled tRNA^His^ we found in each case 100% sequence coverage (Fig. 5c,d, Supplementary Fig. S11). Moreover, all tRNA modifications were identified, which are covered by multiple digestion products. The data confirms that the three bisulphite labels are mapped to position 14, 33 and 55, the expected Ψ-positions provided in the tRNA modification database (Fig. 5e)^40,41^. Finally, we performed the same experiment with tRNA^Leu^, not only to confirm the Ψ-positions but also the loss of the acetyl group of ac^4^C in position 12. Again, the sequence was fully mapped, confirming the correct positions of tRNA alteration (Supplementary Fig. S12).

In summary, we show that bisulphite labelling allows for the quantitative and specific detection of Ψ in synthetic and biological RNA samples by LC–MS/MS at single base resolution. Additionally, numerous other post-transcriptional modifications can be assessed. Even the loss of a modification upon bisulphite labelling was mapped to the correct position, illustrating the profiling of modification dynamics in tRNA.

## Discussion

Ψ is one of the most abundant RNA modifications and has been linked to cellular stress responses (by increase of the RNA half-life), stem cell differentiation and cancer progression^42,43^. Several methods exist for the labelling and detection of Ψ by LC–MS. These methods, however, fail to provide quantitative data and show significant rates of false positives. This provides a major challenge to better understand the dynamics and abundance of Ψ in biological RNAs, which may host multiple Ψs in parallel. With an increasing number of Ψs per sequence, the efficiency of detection is significantly reduced while data analysis becomes problematic.

Here, we report a Ψ-labelling method that allows for the robust and quantitative detection of Ψ and its positional mapping by mass spectrometry in parallel with many other post-transcriptional RNA modifications. We show a near quantitative labelling efficiency, in both synthetic oligonucleotides and isolated yeast tRNAs hosting multiple Ψs simultaneously. In contrast to other LC–MS based labelling detection methods, our analysis did not detect false positives. The workflow requires only low amounts of RNA (a few picomoles), representing a major advantage in contrast to previous labelling strategies. The labelling efficiency in RNAs that contain four or more Ψs is yet to be explored. Given our data we don’t expect a significant drop in labelling efficiency. With the exception of ac^4^C, the labelling method is compatible with all of the tested RNA modifications, but we are currently unable to test all possible modifications for their compatibility with bisulphite labelling. This will be further investigated in the future.

Unlike global quantification of RNA modifications at the nucleoside level from fully digested samples, high-resolution LC–MS/MS offers direct sequence readout of RNA oligonucleotides, providing both sequence context and relative quantification. This approach can distinguish chemical modifications based on their mass, enabling the investigation of modification patterns and interdependencies among multiple RNA modifications. It holds a significant advantage over current sequencing technologies, which are limited to detecting only a few modifications at a time. NGS methods rely on deletion or mutation signatures during reverse transcription for indirect detection of RNA modifications, which can introduce sequence context-dependent biases during library preparation. In contrast, LC–MS/MS allows for a direct mode of detection, minimizing such biases.

However, LC–MS sensitivity decreases for large molecules, limiting the practical oligonucleotide analysis size to approximately 50 nucleotides. This allows short RNAs, such as microRNAs or tRNA fragments (tRFs), to be analyzed without RNase digestion, while longer RNAs like tRNAs or mRNAs require enzymatic digestion to achieve adequate sequence coverage in MS/MS analysis. Notably, LC–MS analysis of mRNA vaccines has highlighted the challenges in achieving full sequence coverage^44^, indicating that transcriptomic analysis with current LC–MS technologies is unlikely to compete with sequencing methods. Nevertheless, LC–MS’s ability to detect multiple modifications simultaneously offers valuable insights into modification patterns and even the discovery of new RNA modifications in short RNA sequences. Realizing this potential will depend on significant advancements in software development to improve analysis and data interpretation.

Although software for the MS/MS analysis of oligonucleotides exists nowadays, the development lacks far behind in comparison with proteomics software solutions. Sequence-dependent analysis of nucleic acid modifications still requires substantial manual effort and is limited to known sequences. The presence of adducts, such as Na^+^ or K^+^, further complicates this process. However, with continued advancements in software solutions, LC–MS-based analysis of short nucleic acid sequences has the potential to become a cost-effective alternative to NGS and third-generation sequencing technologies.

## Methods

### Bisulphite treatment and LC–MS/MS analysis of synthetic RNA oligonucleotides

20 pmols of the RNA oligonucleotide (2 µl of 10 µM stock solution) was mixed with 18 µl of freshly prepared bisulphite reagent (270 mg Na_2_SO_3_ and 34 mg NaHSO_3_ dissolved in H_2_O (total volume: 1 mL, pH 7) and incubated at 70 °C for 4 h. Further, 20 µl (equal volume) of tris–HCl buffer (1.5 M, pH 9) was added to the sample mixture and incubated for 1 h at 37 °C for desulphonation. The oligonucleotides are then desalted from the sample mixture with micro bio spin P6 columns (Bio-Rad) and stored at -20 °C prior to LC–MS analysis.

#### LC–MS/MS analysis

20 µl of the RNA sample is analyzed per injection in a Vanquish HPLC system connected to an Orbitrap Exploris 120 ESI–MS (Thermo Fisher Scientific). The sample components are separated with a flow rate of 0.3 ml/min in a solvent system of 50 mM HFIP (1,1,1,3,3,3,-hexafluoro-2-propanol, 15 mM TEA (triethylamine), pH 9.00 (solvent A) and methanol (solvent B) at 80 °C on DNAPac RP 4 µm column (2.1 × 100 mm, Thermo Scientific). A multistep gradient (3% of solvent B in 0–2 min, followed by an increase from 3 to 10% until 5 min and 10 to 20% until 20 min) was used for separation. MS analysis is performed in the negative mode with a spray voltage of 2500 V, flow of sheath gas, aux gas and sweep gas maintained at 35, 7 and 0 respectively, and ion transfer tube temperature and vaporizer temperature set at 300 °C and 275 °C respectively. For MS1 analysis, the orbitrap resolution of 120,000 was used, the scan range was set from 550 to 3000 m/z, RF lens at 70%, normalized AGC target of 200% and data type set to profile mode. Intensity threshold was set to 1 × e^4^.

For MS/MS analysis, masses of the peaks to be targeted for MS/MS are defined for every run, with a mass tolerance of 3 m/z. The normalized collision energies for CID are set at 15,17 and 19. An orbitrap resolution of 120,000, normalized AGC of 200 and maximum injection time of 400 ms are used. MS/MS data processing is performed in BioPharma Finder v.05.1.

### Quantification of bisulphite labelling

A 30-mer Ψ-containing RNA oligonucleotides and the corresponding 30-mer control RNA (Ψ replaced by U) are mixed in varying ratios (Ψ: 0%, 20%, 40%, 50%, 60%, 80%, 100%). The 100% Ψ sample contains only the RNA with Ψ, while the 0% Ψ sample contains only the control RNA. The final concentration of RNA was maintained at 20 pmols in 20 µl of bisulphite reagent. All samples were treated with bisulphite and analyzed in the LC–MS as described above, with 3 technical replicates. A fitting curve is plotted based on the observed Ψ fraction and the expected Ψ fraction.

### CMC treatment of synthetic RNA oligonucleotides

The experimental method for CMC treatment of oligonucleotides was followed as reported before^37–39^. 1 µg of oligonucleotide is treated with 30 µl of freshly prepared CMC solution (0.17 M CMC, 7 M urea, 4 mM EDTA, 50 mM bicine, pH 8.3) at 37 °C for 20 min. The treatment is stopped with the addition of 100 µl of 0.3 M NaOAc (pH 5.6) and 0.1 M EDTA. 700 µl of chilled ethanol was added to the mixture to precipitate the RNA oligonucleotides, the pellets are further washed twice with 70% ethanol and air dried. The pellets are resuspended in 40 µl of 50 mM Na_2_CO_3_ (pH 10.4) and incubated at 37 °C for 4 h (to remove nonspecific labelling), ethanol precipitated and air dried as before and stored in -20 oC prior to LC–MS analysis. The pellets are resuspended in 80 µl of water, 20 µl of the samples are injected per analysis in the LC–MS. The LC–MS parameters used are described as above with a modified LC gradient. A multistep gradient with 3% of solvent B from 0–2 min, followed by an increase from 3 – 10% until 5 min and 10 to 50% until 20 min was used for separating the different sample components.

### Cyanoethylation of synthetic RNA oligonucleotides

Cyanoethylation of pseudouridine in RNA oligonucleotides was performed as reported before^24^. 2 µg of synthetic Ψ containing RNA is treated with 30 µl of 41% ethanol/1.1.M triethylammonium acetate (pH 8.6) and 4 µl of acrylonitrile and incubated at 70 °C for 2 h. After cyanoethylation, the sample mixture is lyophilized immediately, redissolved in water and the oligonucleotide is recovered by precipitation with 2 M ammonium acetate and 3 vol chilled ethanol. The recovered oligo is dried well, resuspended in 100 µl of water and stored in -20 °C prior to LC–MS analysis. For LC–MS analysis, 20 µl of the treated oligonucleotide is used per injection. The LC–MS parameters used for both CMC treatment and cyanoethylation experiments are the same.

### Bisulphite treatment at acidic pH

EZ RNA methylation kit (Zymo research) was used for the bisulphite treatment of RNA oligonucleotides at acidic pH. 1 µg of the RNA was used for the experiment, which was followed according to manufactures instructions. 1/5^th^ of the final eluted sample was submitted per injection to the LC–MS for analysis. The LC–MS parameters used are the same as used for all other bisulphite treated RNA oligonucleotides.

### Isolation of individual tRNAs

Commercially available yeast tRNA extract (Invitrogen) were used for isolations. Individual tRNAs were isolated from the tRNA extract with biotinylated DNA oligonucleotide probes. 5–10 µl of yeast tRNA extract is mixed with 2 µl of 100 µM biotinylated probe oligonucleotide and 12 µl PEG-400 and 50 µl 1X annealing buffer (20 mM Tris–HCl, pH 7.5, 5 mM MgCl_2_, 0.1 mM DTT, 0.4% Triton X-100), incubated at 90 °C for 5 min for denaturation, followed by a stepwise descend to 25 °C in 45 min for hybridization. To the sample mixture, 50 µl of 5X binding/wash buffer (50 mM Tris HCl (pH 7.5), 5 mM EDTA, 2.5 M NaCl), 100 µl of streptavidin beads (Miltenyi Biotec) and 258 µl of water was added and incubated at 45 °C for 1 h for binding to the microbeads. The magnetically labelled DNA-tRNA complex is passed through the µMACS column following manufacturer’s instructions, followed by washes (3 ml). The tRNA-probe complexes are eluted from the column with preheated (80 °C) elution buffer (10 mM Tris–HCl, pH 7.5, 1 mM EDTA) in 3 stages of 150 µl each, then pooled together and vacuum dried. To generate a larger quantity of tRNAs to aid in optimizations, tRNAs were isolated in multiple rounds. The elutions are pooled together and further HPLC purified to separate the intact tRNA from smaller fragments. The purified tRNAs are lyophilized and resuspended in water, stored at -20 °C for long term storage. The concentration of the resuspended tRNAs is measured with Qubit.

The biotinylated DNA probes used for the isolation of the respective tRNAs are as follows:

- tRNA^His^ (tdbR00000145): [Btn]GGCCACAACGATGTGTACTAACCACTATACTAAGATGGCC.
- tRNA^Arg^ (tdbR00000370): [Btn]TGATTAGAAGTCAGACGCGTTGCCATTACGCCACGCGAGC.
- tRNA^Leu^ (tdbR00000249): [Btn]AGCTTGAATCAGGCGCCTTAGACCGCTCGGCCAAACAACC.

### Bisulphite treatment of isolated tRNAs

50–150 ng of the isolated tRNA is mixed with 20 µl of bisulphite reagent (see above) and incubated at 70 °C for 4 h. Further, 20 µl (equal volume) of tris–HCL buffer (1.5 M, pH 9) was added to the sample mixture and incubated for 1 h at 37 °C for desulphonation. The tRNAs are then desalted from the sample mixture with micro bio spin P6 columns (Bio-Rad) and vacuum dried. The samples are further resuspended in 30 µl of RNase free water and submitted in the LC–MS as a single injection. The LC–MS parameters used are described as above with a modified LC gradient. A multistep gradient starting with 0% of solvent B for 2 min, followed by an increase from 0 – 6% until 20 min, 6 to 10% until 30 min and, 10 to 80% until 35 min was used.

### Bisulphite treatment and RNase T1 digestion of tRNA^His^ and tRNA^Leu^

700—1000 ng of the isolated tRNA is mixed with 20 µl of bisulphite reagent and incubated at 70 °C for 4 h. Further, 20 µl (equal volume) of tris–HCl buffer (1.5 M, pH 9) was added to the sample mixture and incubated for 1 h at 37 °C for desulphonation. The tRNA is then desalted from the sample mixture with micro bio spin P6 columns (Bio-Rad) and vacuum dried prior to RNase digestion. Further, the tRNA is resuspended in 40 µl of digestion buffer and 1.15 µl of 6X diluted RNase T1 beads (Thermo Scientific™ SMART Digest™ RNase Kits) is added to the sample. Mixing and incubation at 37 °C for 15 min allows for partial digestion. The RNase T1 beads are removed from the sample mixture via magnetic separation and the sample mixture is analyzed by LC–MS/MS. As a control, 500 ng of the intact tRNA^His^, without any bisulphite treatment is subjected to RNase T1 digestion and analyzed by the LC–MS/MS.

The RNase digested tRNA fragments were analyzed in the LC–MS as described above with a modified LC gradient. A multistep gradient starting with 0% of solvent B for 2 min, followed by an increase from 0 to 3% until 15 min, 3 to 10% until 40 min and, 10 to 80% until 45 min was used. MS/MS analysis was performed using DDA with a scan range of 200–2000 m/z, with an MS1 resolution of 120,000, a normalized AGC of 200%, an isolation window of 3 m/z, and normalized stepped collision energy of 15,17, and 19.

#### LC–MS/MS Data analysis

All MS/MS data analysis was performed with Biopharma Finder v.05.1 Automatic parameter values were set for component detection. For fragment identification, all MS/MS data was used, with a maximum mass set to 20 kDa, mass accuracy to 10 ppm and minimum confidence to 0.8. Mass search for unspecified modifications were allowed in a mass range of -151 to + 96 Da, with residue deletion allowed. Search for RNase T1 digestion fragments were allowed with a high specificity, limiting the specificity of cleavage at guanosine residues, generating 3’-cyclic phosphate (as most of the fragments generated upon RNase T1 digestion have 3’ cyclic phosphate). Of the identified fragments, only those with a mass error less than 10 ppm, average spectral resolution of 1, and a confidence score of 100% were considered for further analysis.

## Electronic supplementary material

Below is the link to the electronic supplementary material.

Supplementary Material 1

## Data Availability

All LC-MS raw data generated or analyzed during this study is available at: https://owncloud.gwdg.de/index.php/s/n20ombQ3HK3hlSk.
