# Supplementary material for: Quantitative detection of pseudouridine in RNA by mass spectrometry
Source: Sci Rep. 2024 Nov 11;14:27564. doi: 10.1038/s41598-024-78734-3 (PMC11555313; doi:10.1038/s41598-024-78734-3)
Supplement: Supplementary file 1 — Supplementary Material 1 [file 41598_2024_78734_MOESM1_ESM.docx]

Supplementary information for

Quantitative detection of pseudouridine in RNA by mass spectrometry

Shanice Jessica Hermon^1,2^, Anastasia Sennikova^1,2^, Sidney Becker^1,2,*^

^1^ Max-Planck Institute of Molecular Physiology, Dortmund, 44227, Germany ^2^ Department of Chemistry and Chemical Biology, Technical University Dortmund, Dortmund, 44227, Germany * To whom correspondence should be addressed. Email: [sidney.becker@mpi-dortmund.mpg.de](mailto:sidney.becker@mpi-dortmund.mpg.de)

**Supplementary Figure S1.** **Desulphonation for the removal of nonspecific labelling. a,** RNA sequence with two Ψs in parallel (RNA6P) used for tests. **b,** MS1 spectra (M^11-^) of the RNA oligonucleotide prior to any bisulphite treatment showing a single clean peak with a mass of 873.20 and a charge state of 11. **c,** MS1 spectra of the bisulphite treated RNA6P without the desulphonation step, bisulphite labelled peaks are represented as (M + n BS), where n = number of BS labels on the RNA. **d,** MS1 spectra of the bisulphite treated RNA followed by desulphonation, showing a single clean peak with 2 bisulphite labels, with a mass of 888.1 and a charge state of 11.

**Supplementary Figure S2. Test for degradation following bisulphite treatment (pH 7).** Total ion chromatogram (TIC) of bisulphite treated RNA oligonucleotide (RNA4U) prior to any purification. The MS1 data of the oligonucleotide, visible as a single peak in the TIC is shown in the inset. The absence of additional peaks in the TIC proves the absence of degradation of the RNA oligonucleotide following bisulphite treatment.


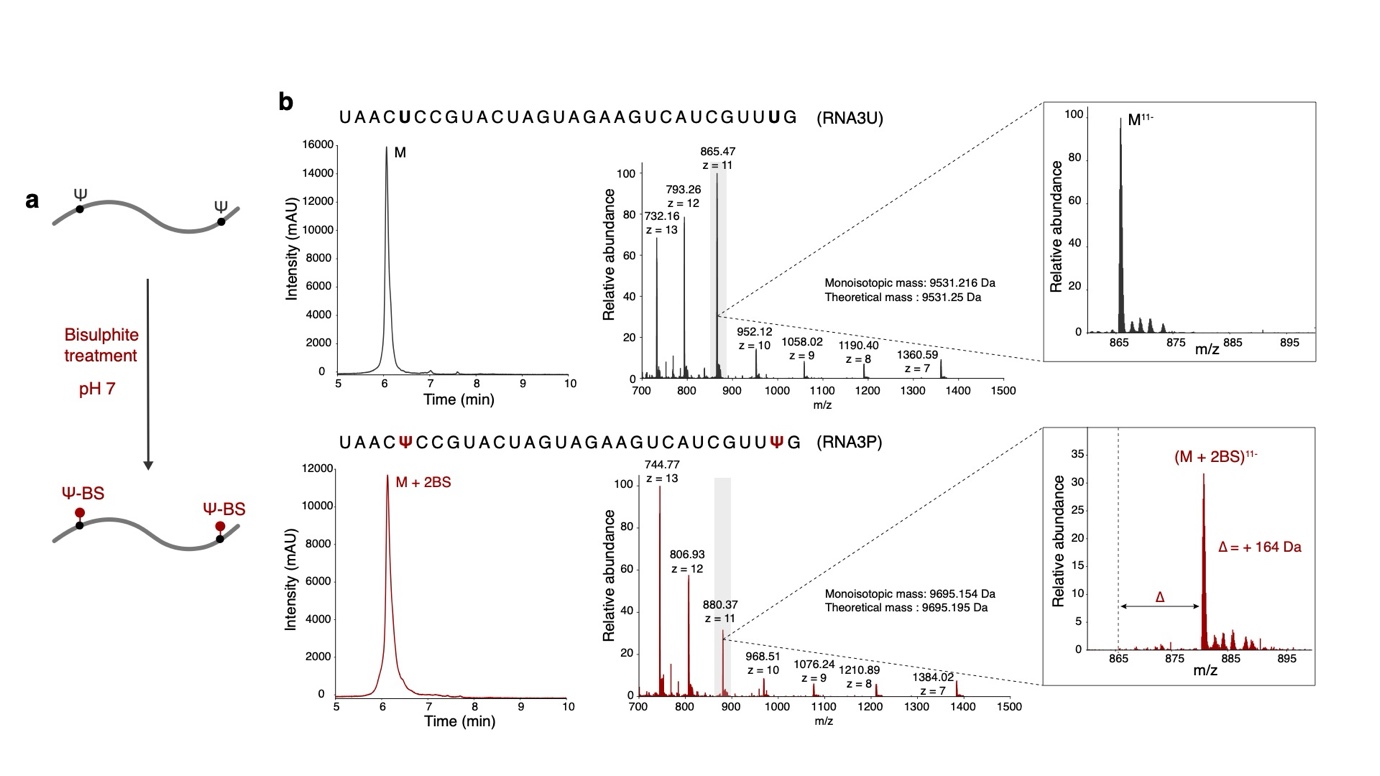

**Supplementary Figure S3. LC-MS/MS analysis of bisulphite treated synthetic RNA oligonucleotide with 2Ψ**

**a,** Schematic representation of the bisulphite treatment of synthetic RNA oligonucleotides RNA3U and RNA3P. **b,** top: HPLC profile (left) and MS1 spectra (right) of the control RNA oligonucleotide (RNA3U) devoid of Ψ, following treatment with bisulphite. The multiple charged negative ion series of the intact RNA3U is shown. The mass peak of 865.47 with a charge state of -11 (M^11-^) is shown in the inset. Bottom: HPLC profile (left) and MS1 spectra (right) of the RNA oligonucleotide hosting two Ψs in parallel (RNA3P), following treatment with bisulphite. The multiple charged negative ion series of the bisulphite labelled intact RNA3P is shown. The mass peak of 880.37, with a charge state of -11 is shown in the inset ((M + 2BS)^13-^). The mass peak of the labelled RNA3P shifts from the control RNA3U (as indicated by the dotted line) by 164 Da. **c,d,** CID spectra of RNA3U and RNA3P treated with bisulphite (sequences mentioned at the top). The precursor ions for fragmentation are M^11-^ and (M+ 2BS)^11-^ respectively. Assignment of the CID fragment ions reveal the positions of the Ψ at 5 and 29, in RNA3P as expected. **e,f,** Representation of all fragment ions identified in both RNA3U and RNA3P following BS treatment.


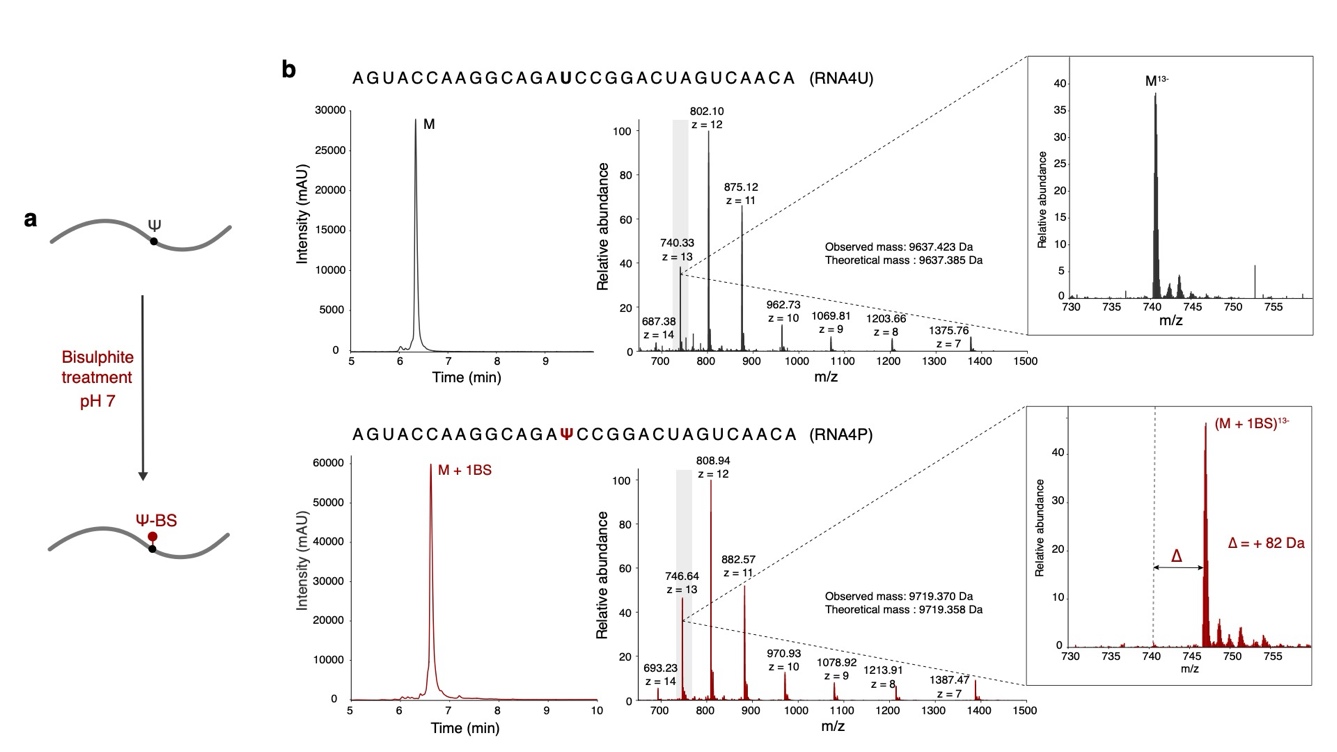

**Supplementary Figure S4. LC-MS/MS analysis of bisulphite treated synthetic RNA oligonucleotide with 1Ψ**

**a,** Schematic representation of the bisulphite treatment of synthetic RNA oligonucleotides RNA4U and RNA4P. **b,** top: HPLC profile (left) and MS1 spectra (right) of the control RNA oligonucleotide (RNA4U) devoid of Ψ, following treatment with bisulphite. The multiple charged negative ion series of the intact RNA4U is shown. The mass peak of 740.25 with a charge state of -13 (M^13-^) is shown in the inset. Bottom: HPLC profile (left) and MS1 spectra (right) of the RNA oligonucleotide hosting one Ψ (RNA4P), following treatment with bisulphite. The multiple charged negative ion series of the bisulphite labelled intact RNA4P is shown. The mass peak of 746.64, with a charge state of -13 is shown in the inset ((M + 1BS)^13-^). The mass peak of the labelled RNA4P shifts from the control RNA4U (as indicated by the dotted line) by 82 Da. **c,d,** CID spectra of RNA4U and RNA4P treated with bisulphite (sequences mentioned at the top). The precursor ions for fragmentation are M^13-^ and (M+ 2BS)^13-^ respectively. Assignment of the CID fragment ions reveal the position of the Ψ at 15 in RNA4P as expected. **e,f,** Representation of all fragment ions identified in both RNA4U and RNA4P following BS treatment.


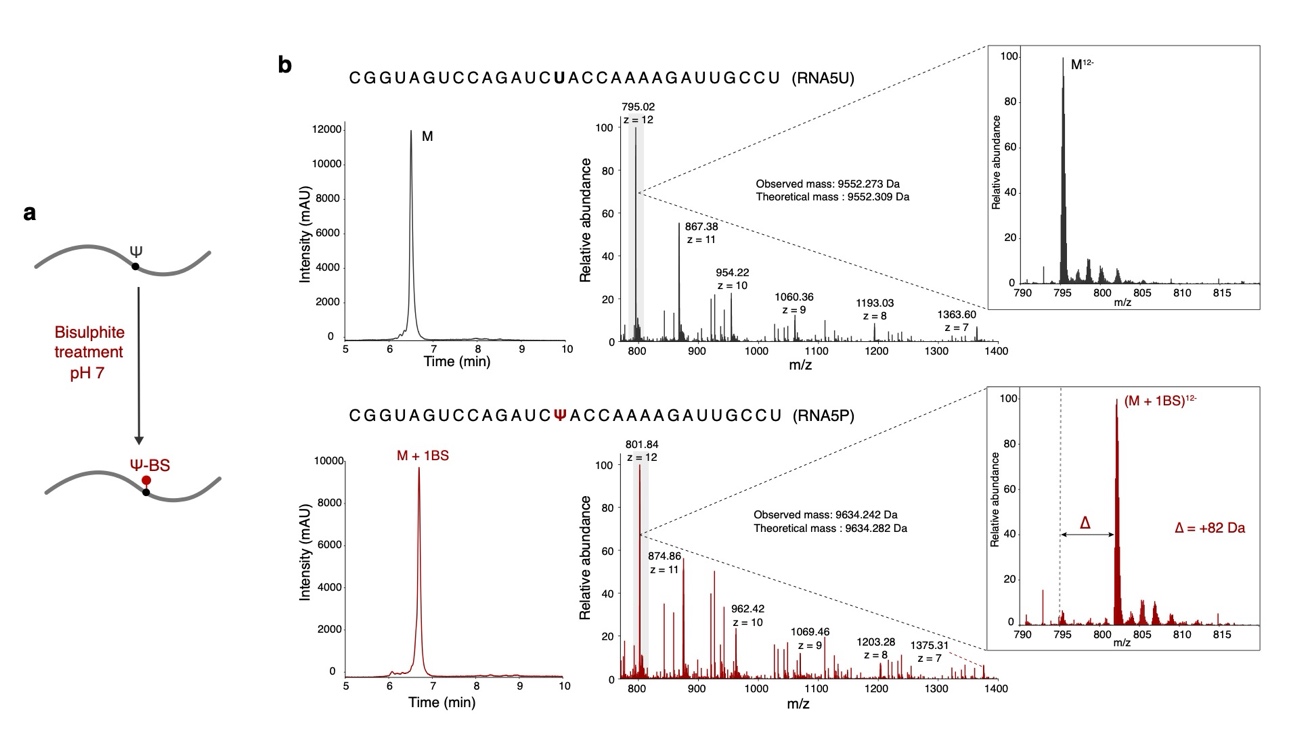

**Supplementary Figure S5. LC-MS/MS analysis of bisulphite treated synthetic RNA oligonucleotide with 1Ψ**

**a,** Schematic representation of the bisulphite treatment of synthetic RNA oligonucleotides RNA5U and RNA5P. **b,** top: HPLC profile (left) and MS1 spectra (right) of the control RNA oligonucleotide (RNA5U) devoid of Ψ, following treatment with bisulphite. The multiple charged negative ion series of the intact RNA5U is shown. The mass peak of 795.02 with a charge state of -12 (M^12-^) is shown in the inset. Bottom: HPLC profile (left) and MS1 spectra (right) of the RNA oligonucleotide hosting one Ψ (RNA5P), following treatment with bisulphite. The multiple charged negative ion series of the bisulphite labelled intact RNA5P is shown. The mass peak of 801.84, with a charge state of -12 is shown in the inset ((M + 1BS)^12-^). The mass peak of the labelled RNA5P shifts from the control (as indicated by the dotted line) by 82 Da. **c,d,** CID spectra of RNA5U and RNA5P treated with bisulphite (sequences mentioned at the top). The precursor ions for fragmentation are M^12-^ and (M+ 2BS)^12-^ respectively. Assignment of the CID fragment ions reveal the position of the Ψ at 15 in RNA5P as expected. **e,f,** Representation of all fragment ions identified in both control and RNA oligonucleotide with Ψ following BS treatment.

**Supplementary Figure S6.** **Sequence context dependency in nonspecific labelling at C and U.**  5 different sequences designed with variable sequence contexts of U and C are treated with bisulphite (pH 7 or acidic pH) to test for nonspecific labelling. No nonspecific labelling is observed in any of the tested sequence contexts when treated with bisulphite at pH 7. The positive control treatment of oligonucleotides with bisulphite at acidic pH (using EZ RNA methylation kit- Zymo Research), shows visible shift in the mass peaks due to the conversion of cytosine bases to uracil.


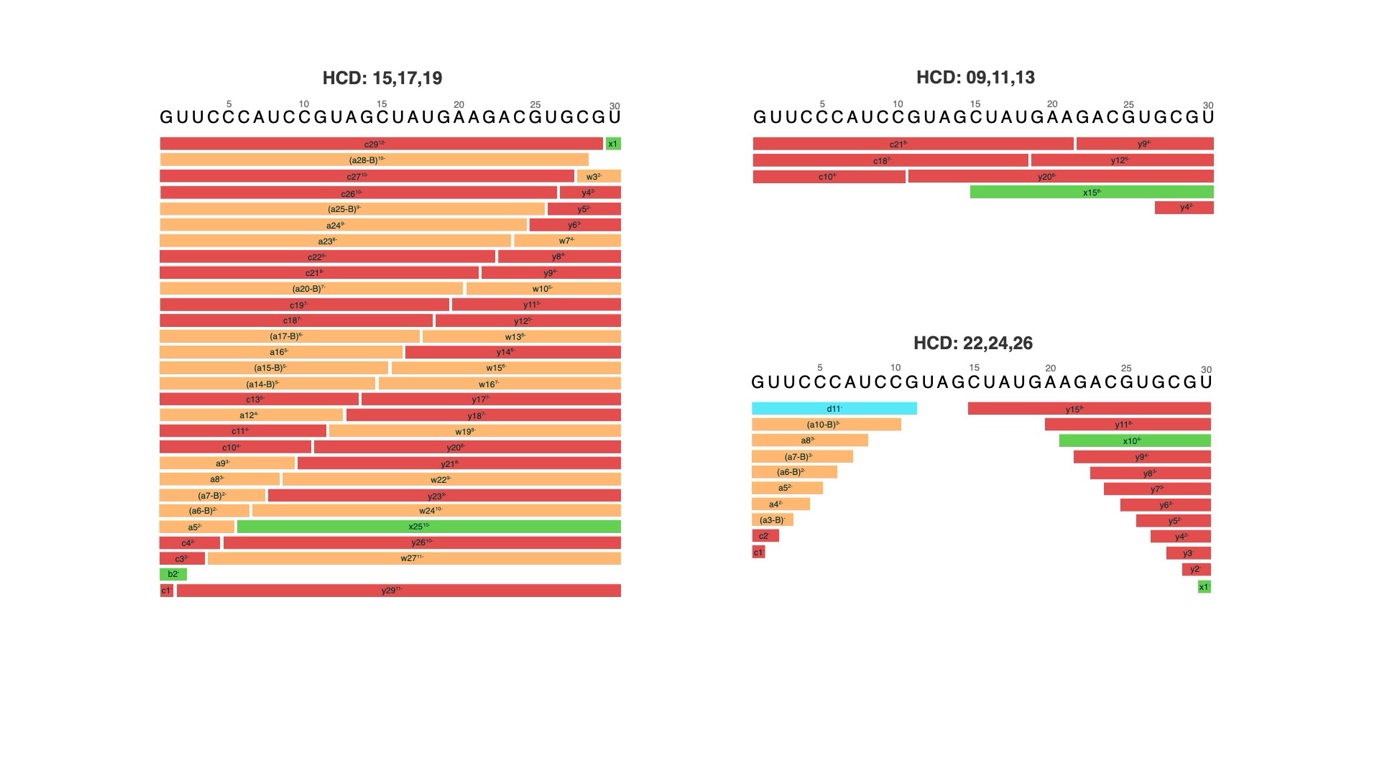


**Supplementary Figure S7.** **Optimizations of MS parameters for improved sequence coverage in MS/MS analysis.** Altering different MS parameters, e.g. higher-energy collisional dissociation (HCD, a type of CID fragmentation), provide different sequence coverage. Conditions were optimized to provide robust sequence coverage not only for 30mers but also for smaller and larger fragments to allow for the analysis of RNase T1 digested tRNAs.

**Supplementary Figure S8.** **Comparison of the specificity and efficiency of labelling Ψ by CMC, acrylonitrile and bisulphite**.

**a,** RNA sequence with two Ψs in parallel (RNA7P) used for all three labelling studies. **b,** HPLC profile of the bisulphite labelled RNA7P showing a single clean peak and the chemical structure of Ψ-BS adduct (top). MS1 spectra of the BS labelled RNA7P showing a single clean peak with a mass of 811.59, with a charge state of -12 (bottom). **c,** HPLC profile of the CMC labelled RNA7P, and the chemical structure of the CMC labelled Ψ (top). MS1 spectra of the CMC labelled RNA, the mass peak of the intact mass of the RNA is labelled as M^12-^, CMC labelled peaks are represented as (M + n CMC) ^12-^, where n = number of CMC labels on the RNA. **d,** HPLC profile of the cyanoethylated (ce) RNA7P, and the chemical structure of the acrylonitrile labelled Ψ. (top). MS1 spectra of the acrylonitrile labelled RNA7P, the mass peak of the intact mass of the RNA is labelled as M^12-^, cyanoethylated peaks are represented as (M+ n ce)^12-^, where n = number of acrylonitrile labels on the RNA.


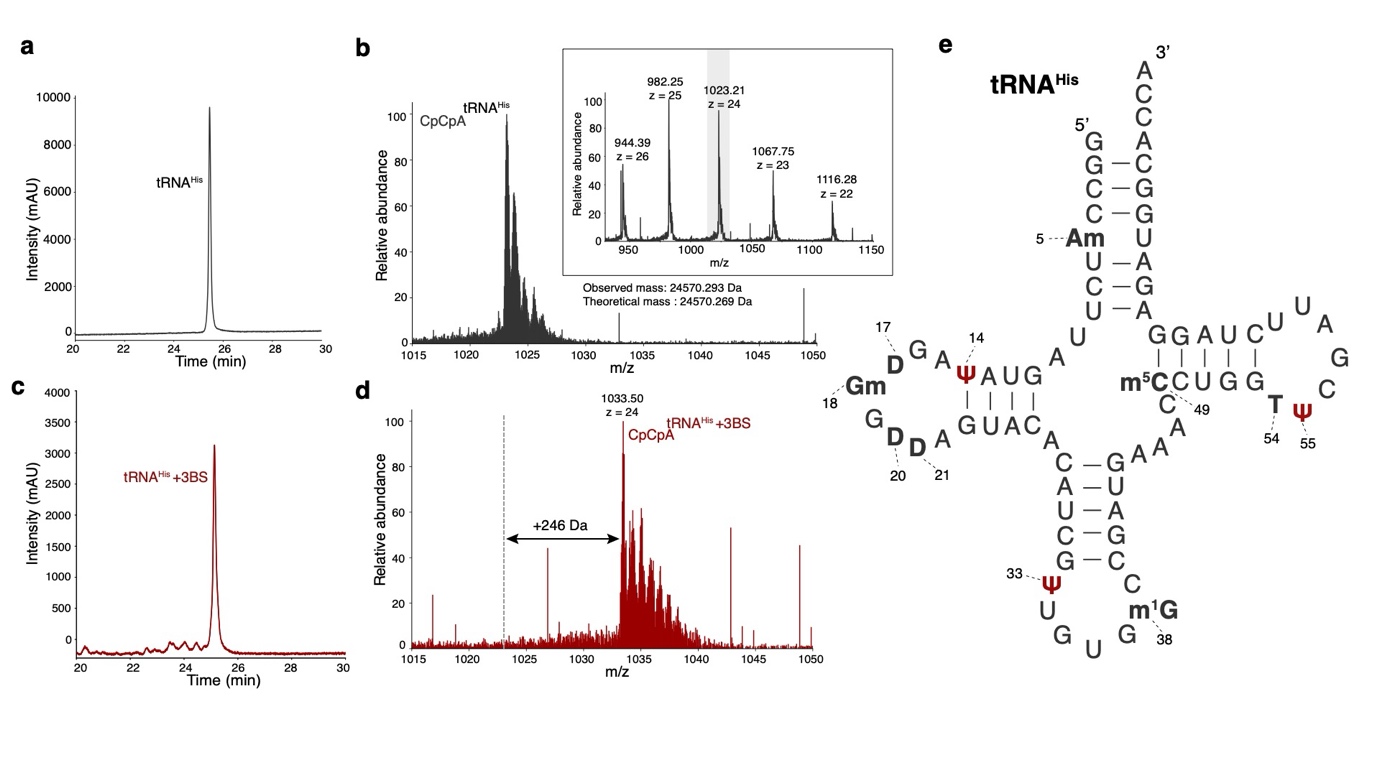


**Supplementary Figure S9. LC-MS analysis of bisulphite labelled tRNA^His^: a,b,** LC-MS analysis of the intact tRNA^His^. UV chromatogram at 260 nm showing the single isolated peak of tRNA^His^ (left), intact mass analysis of the isolated tRNA^His^ (right). The mass peak of 1023.23, with a charge state of -24, is shown. The series of multiply charged negative ions of the intact tRNA^His^ is shown in the inset. **c,d,** LC-MS analysis of the bisulphite labelled intact tRNA^His^. The UV chromatogram at 260 nm (left), and the mass peak at 1033.50, with a charge state of -24 is shown. The mass peak of the labelled tRNA^His^ shifts from the unlabeled one (as indicated by the dotted line) by 246 Da. **e,** Secondary structure of yeast tRNA^His^ (database ID: tdbR00000145)^1,2^. The positions of all the modified residues identified is marked. The symbols for the modified nucleosides are as follows: Am, 2'-O-methyladenosine; D, Dihydrouridine; Psi, Pseudouridine; Gm, 2'-O-methylguanosine; m^1^G, 1-methylguanosine; m^5^C, 5-methylcytidine; T, Thymidine.


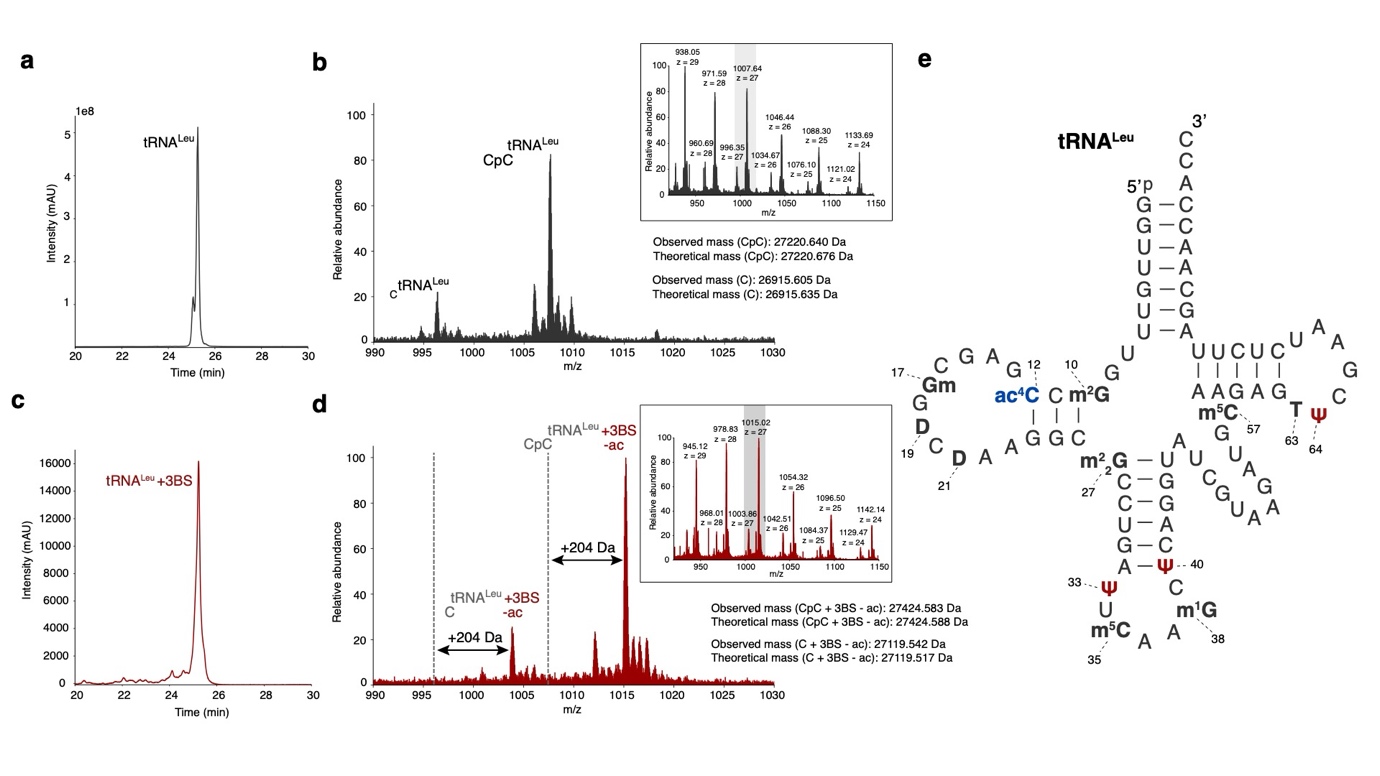


**Supplementary Figure S10. LC-MS analysis of bisulphite labelled tRNA^Leu^: a,b,** LC-MS analysis of the intact tRNA^Leu^. UV chromatogram at 260 nm showing the isolated peaks of tRNA^Leu^ (left), intact mass analysis of the isolated tRNA^Leu^ (right) The mass peaks of 1007.64, and 996.35 with charge states of -27, of the unlabeled intact tRNA is shown. The series of multiply charged negative ions of the intact tRNA^Leu^ is shown in the inset **c,d,** LC-MS analysis of the bisulphite labelled intact tRNA^Leu^. The UV chromatogram at 260 nm (left), and the mass peaks at 1015.02 and 1003.86, with a charge state of -24 is shown. The mass peak of the labelled tRNA^Leu^ shifts from the unlabeled one (as indicated by the dotted line) by 204 Da This corresponds to the mass of three bisulphite labels at Ψ and deacetylation at ac^4^C (246 Da – 42 Da = 204 Da). **e,** Secondary structure of yeast tRNA^Leu^ (Leu tdbR00000249)^1,2^. The positions of all the modified residues identified is marked. The symbols for the modified nucleosides are as follows: m^2^G, N2-methylguanosine; ac^4^C, N4-acetylcytidine; Gm, 2'-O-methylguanosine; D, Dihydrouridine; m^2^,_2_G, N2,N2-dimethylguanosine; Ψ, Pseudouridine; m^5^C, 5-methylcytidine; m^1^G, 1-methylguanosine; T, Thymidine.


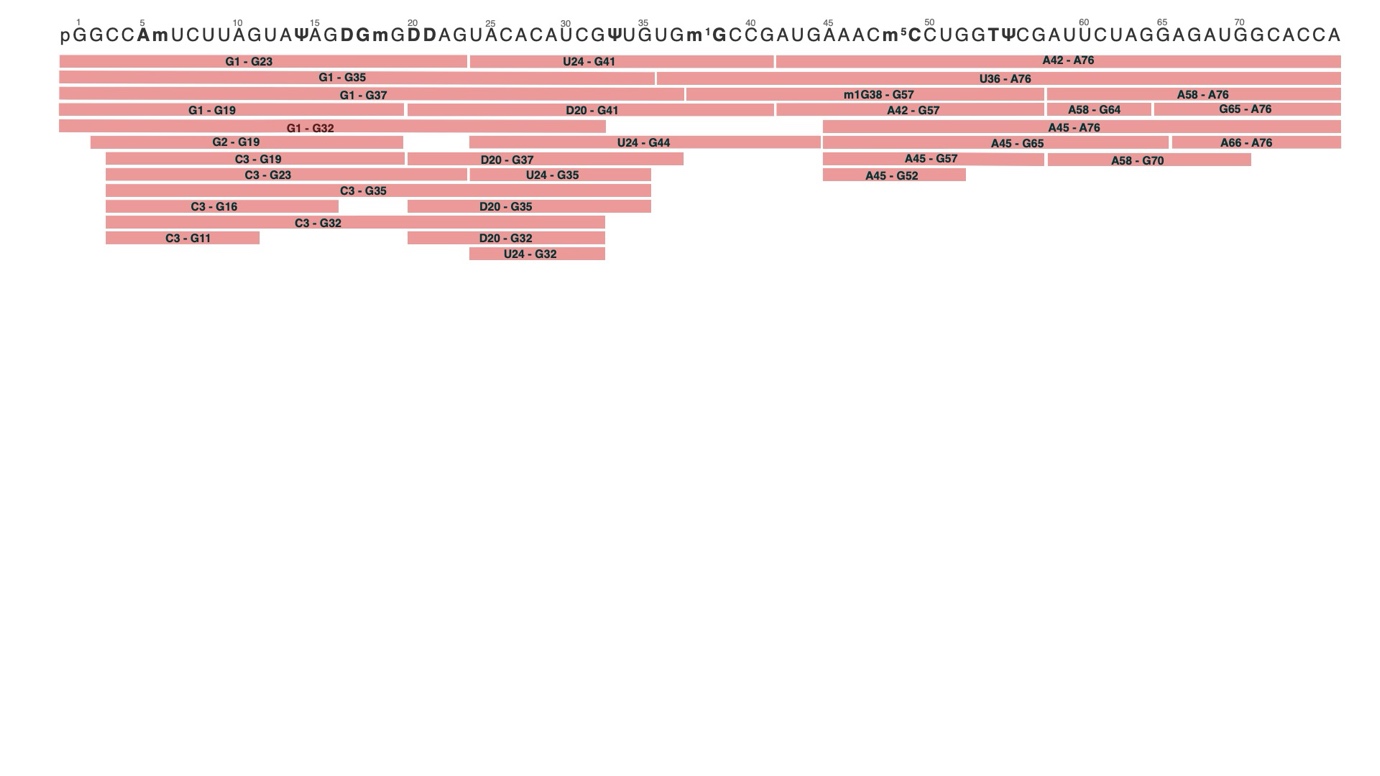


**Supplementary Figure S11. Sequence map of the RNase T1 digested unlabeled tRNA^His^:** Sequence map showing all identified RNase T1 digestion fragments from the unlabeled tRNA^His^. Only fragments with a 2’3’-cyclic phosphate, with an average spectral resolution of 1, confidence of 100 and error < 5 ppm are considered for sequence mapping. The sequence map covers 100% of the tRNA^His^ sequence identifying all expected modifications as from the database.


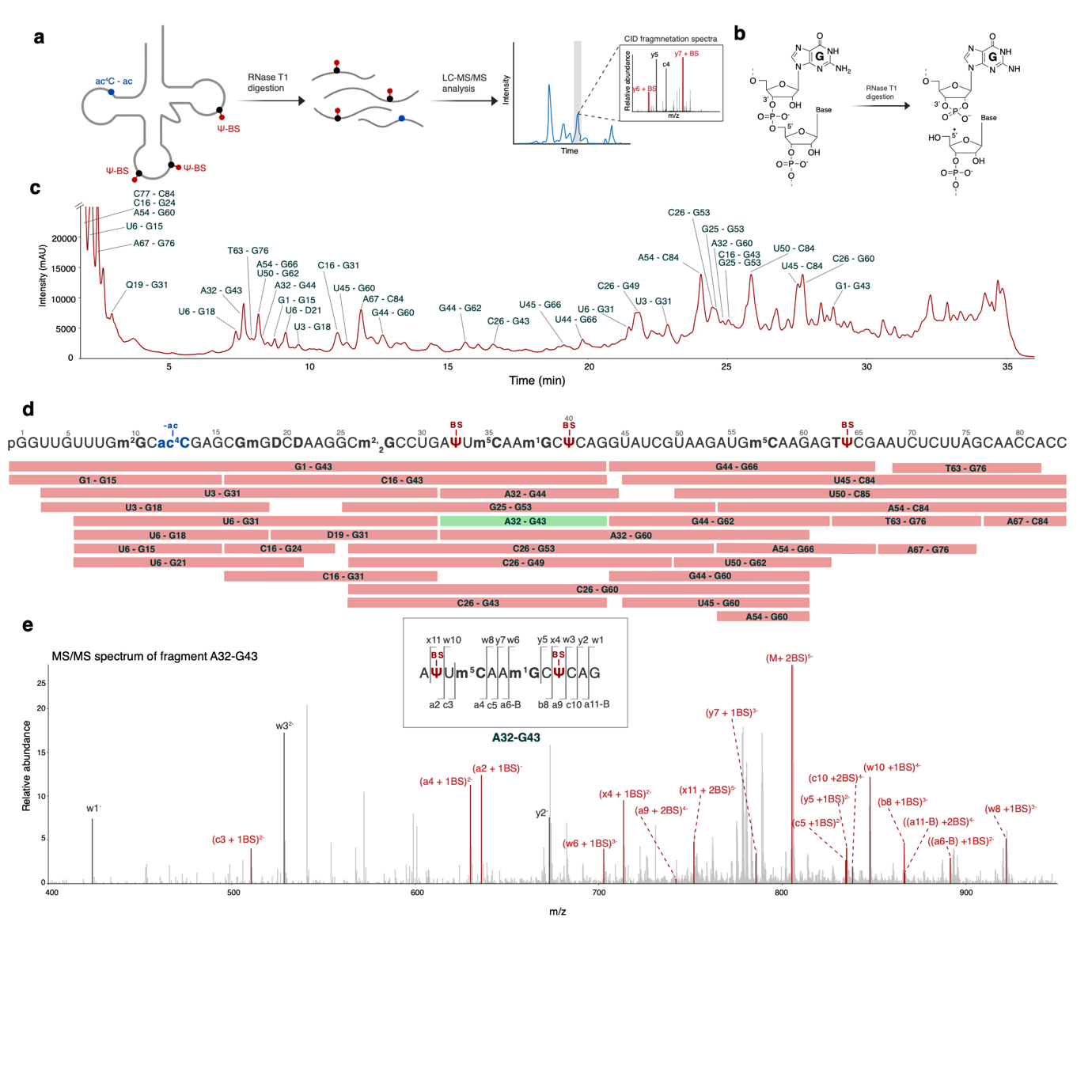


**Supplementary Figure S12. LC-MS/MS analysis of bisulphite labelled tRNA^Leu^ following digestion with RNase T1**

**a,b,** Schematic representation of RNase T1 digestion of tRNA^Leu^ and LC-MS/MS analysis. RNase T1 cleaves after guanosine, generating 5’-OH at the adjacent nucleoside residue and 2’3’-cyclic phosphate termini at the guanosine^3^ **c,** HPLC profile of the RNase T1 digested fragments of the bisulphite labelled tRNA^Leu^, all identified fragments are labelled. **d,** Sequence map showing all MS/MS identified RNase T1 digestion fragments from the bisulphite labelled tRNA^Leu^. Only fragments with a 2’3’-cyclic phosphate, full structural resolution and error < 10 ppm are considered for sequence mapping. The sequence map covers 100% of the tRNA^Leu^ sequence identifying all expected modifications including labelled Ψs and deacetylated ac^4^C. **e,** CID spectra of the fragment A32 – G43. The precursor ion for fragmentation is (M+ 2BS)^5-^.

**Supplementary Table S1. Data used to analyze the quantitative nature of bisulphite labelling.** The values for relative abundance of Ψ/U, and sum intensity of Ψ/U are directly acquired following analysis in the BioPharma Finder v5.1 (Thermo Fisher Scientific). Detected fractions of Ψ are calculated from the relative abundance of Ψ and used to plot the fitting curve.

| **Theoretical ratio of Ψ : U** | **Relative abundance of Ψ** | **Sum intensity of Ψ** | **Relative abundance of U** | **Sum intensity of U** | **Detected fraction of Ψ** | **Detected fraction of U** |
| --- | --- | --- | --- | --- | --- | --- |
| 0:100 | 0 | 0 | 100 | 26200000 | 0 | 100 |
| 0:100 | 0 | 0 | 100 | 29300000 | 0 | 100 |
| 0:100 | 1.42 | 305000 | 100 | 21600000 | 1.4 | 98.59 |
| 20:80 | 19.17 | 910000 | 100 | 4750000 | 16.09 | 83.91 |
| 20:80 | 18.01 | 931000 | 100 | 5170000 | 15.26 | 84.73 |
| 20:80 | 19.06 | 12000000 | 100 | 62700000 | 16.01 | 83.99 |
| 40:60 | 50.05 | 1950000 | 100 | 3890000 | 33.35 | 66.64 |
| 40:60 | 49.46 | 2050000 | 100 | 4150000 | 33.09 | 66.9 |
| 40:60 | 57.30 | 2650000 | 100 | 2650000 | 36.42 | 63.57 |
| 50:50 | 84.13 | 26300000 | 100 | 31300000 | 45.69 | 54.30 |
| 50:50 | 72.35 | 4330000 | 100 | 5980000 | 41.97 | 58.02 |
| 50:50 | 80.85 | 20800000 | 100 | 25700000 | 44.7 | 55.29 |
| 60:40 | 100 | 2500000 | 82.05 | 2050000 | 54.92 | 45.07 |
| 60:40 | 100 | 1360000 | 76.01 | 1040000 | 56.81 | 43.18 |
| 60:40 | 100 | 13500000 | 59.89 | 8060000 | 62.54 | 37.45 |
| 80:20 | 100 | 49400000 | 19.77 | 9770000 | 83.49 | 16.5 |
| 80:20 | 100 | 6030000 | 35.51 | 2140000 | 73.79 | 26.2 |
| 80:20 | 100 | 9580000 | 36.25 | 3470000 | 73.39 | 26.6 |
| 100:0 | 100 | 53100000 | 1.66 | 879000 | 98.36 | 1.63 |
| 100:0 | 100 | 13900000 | 1.03 | 143000 | 98.98 | 1.02 |
| 100:0 | 100 | 8680000 | 2.26 | 196000 | 97.78 | 2.21 |

References

1. Sajek, M. P., Woźniak, T., Sprinzl, M., Jaruzelska, J. & Barciszewski, J. T-psi-C: user friendly database of tRNA sequences and structures. *Nucleic Acids Res* (2019) doi:10.1093/nar/gkz922.

2. Dunin-Horkawicz, S. MODOMICS: a database of RNA modification pathways. *Nucleic Acids Res* **34**, D145–D149 (2006).

3. Pace, C. N., Heinemann, U., Hahn, U. & Saenger, W. Ribonuclease T1: Structure, Function, and Stability. *Angewandte Chemie International Edition in English* **30**, 343–360 (1991).
